# The Spectrum of Cutaneous Adverse Drug Reactions Following the Application of Topical Medications: An Observational Study at a Tertiary Care Center

**DOI:** 10.7759/cureus.28139

**Published:** 2022-08-18

**Authors:** Vaishali S Makwana, Sejal G Bhadja, Bhavesh M Songara, Zalak R Patel, Aniruddha P Vyas

**Affiliations:** 1 Department of Dermatology, C. U. Shah Medical College, Surendranagar, IND

**Keywords:** over the counter cream, triple combination, fixed drug combination, tinea incognito, cutaneous adverse drug reaction

## Abstract

Introduction

Topical medications are one of the most commonly used therapeutic agents in treating a wide spectrum of dermatological diseases. The misuse of topical medicines for inappropriate indications and an extended period may result in cutaneous adverse drug reactions (ADR).

Aims

We conducted this study to observe demographic parameters, commonly misused topical medicines, various clinical patterns of cutaneous ADR, and source of drug prescription among study participants.

Materials and methods

This cross-sectional observational study was conducted from October 2021 to May 2022 at the dermatology outpatient department (OPD) of a tertiary care center. All patients who presented with worsening pre-existing skin diseases or the development of skin disease after the topical application of some cream or ointment were included in the study with written informed consent. A detailed history was taken, and a clinical examination was done.

Results

We detected 200 cases (1.62%) of cutaneous ADR out of 12,346 OPD patients in the eight-month study period. The most common age group was 21-30 years (30%). Most of the patients had used topical medicines for fungal infections (76%). The most commonly used topical medication was a fixed drug combination (FDC) of steroid, antifungal, and antibacterial agents (40%). Tinea incognito (36%) was the most common cutaneous ADR noted.

Conclusion

This study shows that misuse of topical medications is rampant in our community because of their free and easy availability. There is an urgent requirement for strict regulations over the manufacturing, sales, and marketing of over-the-counter (OTC) topical medications to reduce the incidence of cutaneous ADR.

## Introduction

Topical medications are fundamental in dermatological therapeutics, offering the ability to deliver treatment directly to the skin while limiting systemic side effects [1]. The choice of topical medications depends on the nature of the disease, site affected, age and sex of the patients, vehicle, frequency, and duration of application to avoid cutaneous adverse drug reaction (ADR); for this reason, topical medications need to be prescribed by dermatologists only for their correct indications. Misuse of topical medicines for inappropriate indications may result in cutaneous adverse drug reactions.

A cutaneous adverse drug reaction is defined as any undesirable change in the structure or function of the skin, its appendages, or mucous membranes caused by a drug. It encompasses all adverse events related to drug eruption, regardless of etiology [2]. Here, we have conducted this study to observe various cutaneous adverse drug reactions following the application of topical medications.

People generally use over-the-counter (OTC) topical medications for common skin conditions like acne, hyperpigmentation, hypopigmentation, bacterial and fungal infections, eczema, pruritus, burns, etc. OTC or nonprescription medicine is the medicine that anyone can buy without a prescription from a health care professional [3]. Commonly available OTC creams or ointments for skin conditions include steroids, antibiotics, antifungals, dithranol, coal tar, salicylic acid, urea, hydroquinone, tretinoin, herbal ingredients, various fixed drug combination (FDC) creams, etc.

There are very few studies in India addressing this problem [3-5]. We have conducted this study to observe demographic data, various clinical presentations of cutaneous adverse drug reactions, commonly used agents, mode of misuse, and source of drug prescription. There is a need to raise awareness about hazardous effects on the skin due to the free availability of these topical medications.

## Materials and methods

Study design and ethical approval

A cross-sectional observational study was conducted at C. U. Shah Medical College and Hospital, Surendranagar. Ethical approval was obtained from the Institutional Ethics Committee of C. U. Shah Medical College with reference no. CUSMC/IEC(HR)/PUB-28/2022/Final Approval/125/2022.

Inclusion and exclusion criteria

This study was carried out at the dermatology outpatient department (OPD) of a tertiary care center between October 2021 and May 2022. Out of the 12,346 OPD patients in this duration, 200 patients presenting with cutaneous adverse drug reactions following the use of topical medications were enrolled in the study after written informed consent. We included patients of both sexes and all age groups in this study. In patients less than 12 years of age, the consent of the parents was taken. Those patients with comorbidities that could cause similar changes to those of the side effects produced by topical medication were excluded from the study. For example, Cushing's disease, systemic lupus erythematosus, peripheral vascular disease, etc.

Methodology

History was taken using a detailed questionnaire including demographic parameters, names of topical agents, indications of use, duration and frequency of application, source of the drug (prescribed by dermatologists, non-dermatologist clinicians, pharmacists, relatives, or self-application), symptoms, etc. Photographs were taken, and cutaneous and systemic examination was done.

Statistical analysis

Data were entered in Microsoft Excel (Microsoft Corporation, Redmond, WA) and results were analyzed using Epi info (Version 7.1.5) from the Centers for Disease Control and Prevention (CDC, Atlanta, USA).

## Results

This study reveals that the proportion of cutaneous ADR after misuse of topical medications was 1.62% (200/12,346 patients). In the present study, a total of 200 patients aged from four to 82 years were included. Most cases belonged to the 21 to 30 years age group (30%) followed by 11 to 20 years (21%). The study population comprised 104 females (52%) and 96 males (48%). Out of 200 cases, 69.5% cases (n=139) were literate. According to occupational history, housewives were most commonly affected (32%), followed by manual workers (31.5%). 54% of patients belonged to rural areas. The duration of usage varied from one day to one year. Most cases (49%) had used these creams for a duration of one to three months. Very few (1%) patients had used topical medication for more than six months. Large numbers of patients (55%) applied the cream twice a day (Table 1).

**Table 1:** Demographic details of patients presenting with cutaneous adverse drug reactions following the application of topical medications

Parameters	n=200 (%)
Most common Age group	
21-30 years	60 (30%)
Gender	
Male	96 (48%)
Female	104 (52%)
Education	
Illiterate	61 (30.5%)
Literate	139 (69.5%)
Occupation	
Student	51 (25.5%)
Housewife	64 (32%)
Manual worker	63 (31.5%)
Office worker	14 (7%)
Non-working (retired, children)	8 (4%)
Residence	
Urban	92 (46%)
Rural	108 (54%)
Duration of usage	
< 1 month	73 (36.5%)
1-3 months	98 (49%)
3-6 months	27 (13.5%)
6-12 months	2 (1%)

The most common indication for which topical medications were used was fungal infection (76%; n=152) followed by melasma (11.5%; n=23), and pruritus (3.5%; n=7) (Table 2).

**Table 2:** Indications of using topical medications by the patients

Indication	n= 200 (%)
Fungal infections (Dermatophytosis, Candidiasis, Pityriasis Versicolor)	152 (76%)
Melasma	23 (11.5%)
Pruritus	7 (3.5%)
Viral infections (Wart, Molluscum Contagiosum)	5 (2.5%)
Psoriasis	4 (2%)
Acne	4 (2%)
Bacterial infections (Pyoderma)	2 (1%)
Eczema	1 (0.5%)
Scabies	1 (0.5%)
Burn	1 (0.5%)

Most commonly used cream was combination of steroid + antifungal+ antibacterial (fixed drug combination) in 40% (n=80) of cases, followed by triple combination containing (steroid + hydroquinone + tretinoin) in 15% of patients (n=30) (Table 3).

**Table 3:** Various types of topical medications implicated in reported cutaneous adverse drug reactions

Topical medications	n =200(%)
Fixed dose combination - (Steroid + antibacterial + antifungal)	80 (40%)
Clobetasol propionate 0.05% + Gentamicin sulphate 0.1% + Miconazole nitrate 2%	25
Clobetasol propionate 0.05% + Neomycin sulphate 0.5% + Miconazole nitrate 2%	20
Clobetasol propionate 0.05% + Neomycin sulphate 0.5% + Ketoconazole 2%	13
Clobetasol propionate 0.05% + Ofloxacin 0.75% + Ornidazole 1% + Itraconazole 1%	9
Beclomethasone dipropionate 0.025% + Neomycin sulphate 0.5% + Econazole nitrate 0.1%	6
Beclomethasone dipropionate 0.025% + Neomycin sulphate 0.5% + Miconazole nitrate 2%	5
Clobetasol propionate 0.05% + Ciprofloxacin hydrochloride 1% + Terbinafine hydrochloride 1%	2
Triple combination	30 (15%)
Mometasone furoate 0.1% + Hydroquinone 2% + Tretinoin 0.025%	30
Steroid + antibacterial	7 (3.5%)
Betamethasone dipropionate 0.05% + Gentamicin sulphate 0.1%	7
Steroid + antifungal	18 (9%)
Beclomethasone dipropionate 0.025% + Clotrimazole 1%	18
Steroid	3 (1.5%)
Clobetasol propionate 0.05%	3
Antifungal	5 (2.5%)
Luliconazole 1%	5
Antibacterial	2 (1%)
Neomycin sulfate 0.05%	2
Other over-the-counter products	55 (27.5%)
Sapat lotion, Derobin, salicylic acid, coal tar, *malam* (Zalim, Skin Sudha, Ayushi, Parasmani, Soraheal, Dharam, KB Ghoram or Mahendra Patel)	55

**Figure 1:**
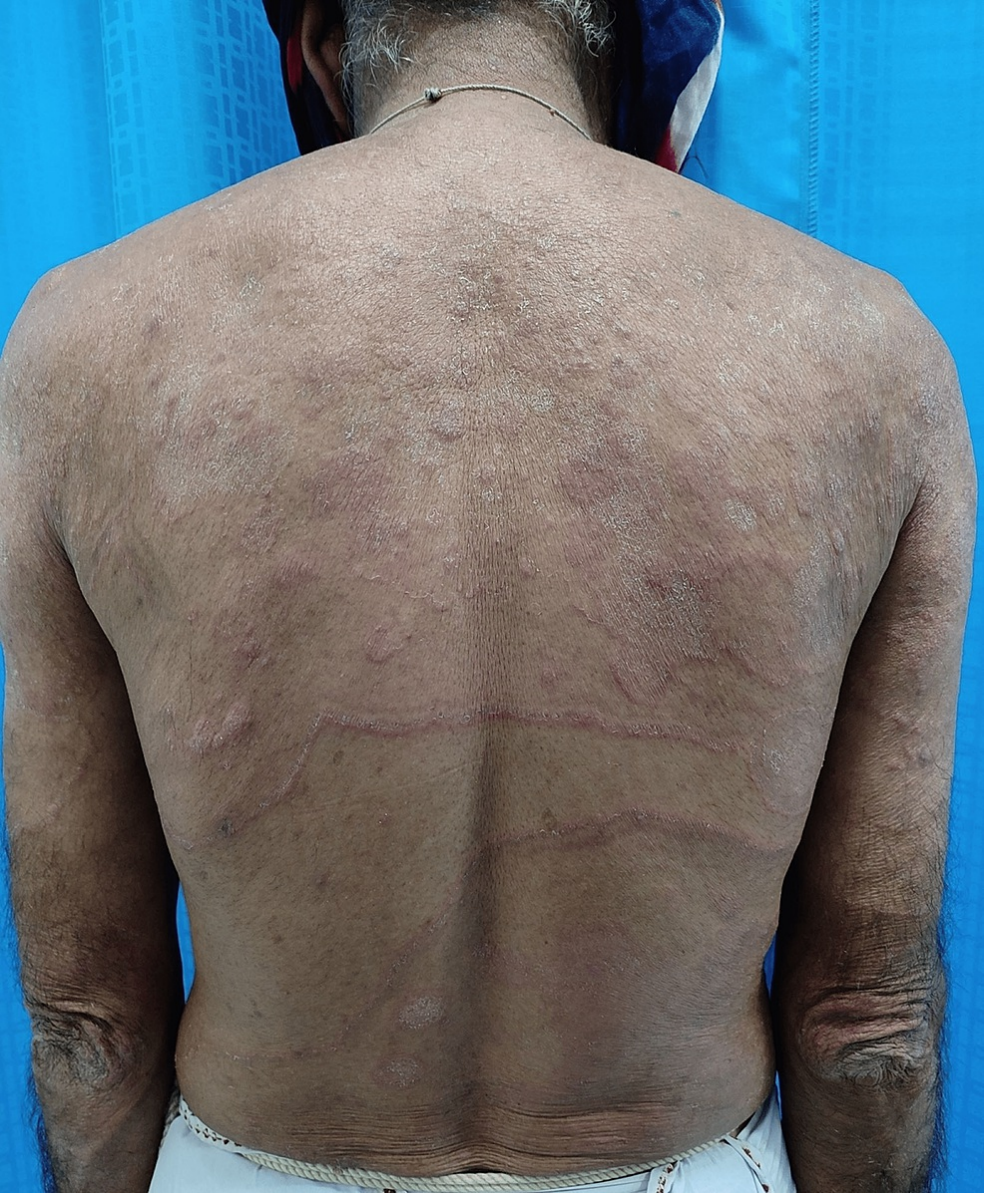
Tinea incognito

**Figure 2:**
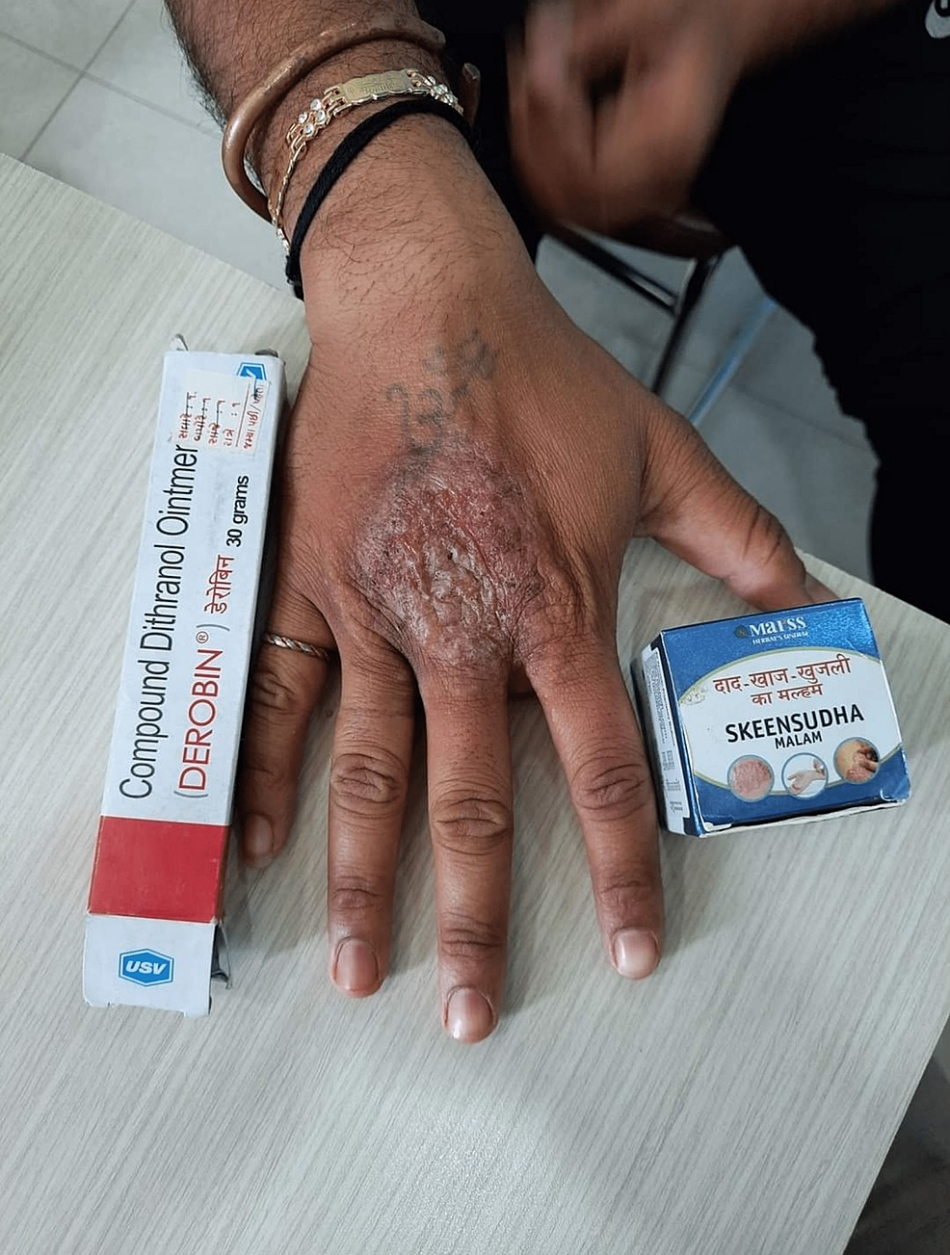
Irritant contact dermatitis

Tinea incognito (36%) (Figure 1) was the most common entity of cutaneous ADR, followed by irritant contact dermatitis in 12.5% (Figure 2) cases.

The proportions of various types of cutaneous ADR are mentioned in Table 4.

**Table 4:** Clinical patterns and the most common causative agents for cutaneous adverse drug reactions

Cutaneous adverse drug reactions	n=200 (%)	Agents (Most common)
Tinea incognito	72 (36%)	Fixed drug combination
Irritant contact dermatitis	25 (12.5%)	Sapat lotion
Eczematous changes	23 (11.5%)	Parasmani Malam
Striae rubra	19 (9.5%)	Fixed drug combination
Hyperpigmentation	18 (9%)	Fixed drug combination
Acneiform eruption	16 (8%)	Triple combination
Telangiectasia	9 (4.5%)	Triple combination
Secondary bacterial infection	4 (2%)	Fixed drug combination
Chemical burn	4 (2%)	Derobin cream
Hypertrichosis	3 (1.5%)	Triple combination
Photosensitivity	3 (1.5%)	Parasmani Malam
Hypopigmentation	3 (1.5%)	Triple combination
Necrotic change	1 (0.5%)	Zalim Malam

Out of 200 cases, the majority of patients (43%) purchased these creams directly from pharmacists without a prescription. 29.5% of people did self-medication, and 1.5% of patients used prescribed cream from dermatologists, which they used beyond the advised period (Table 5). 

**Table 5:** Sources of topical medications prescription

Sources	n =200(%)
Pharmacist	86 (43%)
Self-medication	59 (29.5%)
Non dermatologist doctor	36 (18%)
Relative	16 (8%)
Dermatologist	3 (1.5%)

Comparative analysis with other published studies was done (Table 6).

**Table 6:** Comparison of data of present study with published studies ADR: adverse drug reaction; n: number of patients

	Our Study	Birudala et al. [3]	Dey et al. [4]	Swaroop et al. [5]	Meena et al. [6]	Dabas et al. [7]
Year	2021-2022	2017-2018	2010-2011	2018-2019	2015-2016	-
Study period	8 months	1 year	1 year	1 year	1 year	-
Total number of patients (n)	200	453	379	100	370	100
Most common age group (in years)	21-30 (30%)	21-30 (50.3%)	20-29 (37.73%)	21-30 (35%)	11-20 (39.73%)	19-45 (74%)
Gender						
Male	48%	35.76%	21.11%	28%	62.70%	75%
Female	52%	64.24%	78.89%	72%	37.3%	25%
Source of drug (Most common)	Pharmacist (43%)	-	Pharmacist (35.36%)	Friends (46%)	Pharmacist (34.86%)	Pharmacist (30.88%)
Most common Indication of use	Fungal infection (76%)	Acne (39.7%)	Lightening of skin (50.39%)	Acne (35%)	Dermatophytosis (52.43%)	Tinea cruris (44%)
Duration of use (Most common)	1-3 months (49%)	-	<1 year (69.39%)	>1 year (29%)	1 week-1 month (48.91%)	-
Most common Adverse effect	Tinea incognito (36%)	Acneiform eruption (51.6%)	Facial acne (37.99%)	Acneiform eruption (45.2%)	Tinea incognito (49.46%)	Burning sensation (44.18%)
Most common Topical medication	Antifungal+ antibacterial+ steroid (40%)	Clobetasol propionate (41.1%)	Antifungal+ antibacterial+ steroid (39.84%)	Betamethasone valerate (32%)	Clobetasol propionate (44.32%)	Steroid based preparation (clobetasol propionate) (40%)
Proportion of cutaneous ADR	1.62%	-	5.63%	-	0.43%	-

## Discussion

Topical medications provide effective treatment for various dermatological disorders. They provide drug delivery directly to the skin, reducing the risk of systemic side effects. Misuse of these drugs by non-dermatologist doctors, pharmacists, and patients for quick relief of skin diseases has increased the proportion of cutaneous ADR.

The age of the study population ranged from four years to 82 years. The most common age group affected was 21-30 years (30%) (n=60). This finding was similar to the study conducted by Dey et al. (20-29 years, 37.73%) [4] and Saraswat et al. (21-30 years, 36%) [8]. The younger age group is more cosmetically conscious. They believe that these topical medications will provide rapid clearing of their skin diseases. Apart from this, frequent use of the internet to find treatment remedies is prevalent in the young population.

In our study, a female preponderance was seen. The male to female ratio (M: F) was 0.9:1. Our finding was similar to that of the study conducted by Birudala et al. (M: F = 0.6:1) [3]. Over-the-counter, freely available fairness creams are responsible for the raised numbers of female patients in our study. In contrast, research conducted by Varshney et al. showed a male preponderance with an M: F ratio of 1.2:1 [9].

In the current study, the majority of patients were educated up to secondary level and above 44.5% (n=89) contrary to the study by Thomas et al., which showed that the most patients were educated till primary education (31.3%) [10].

In our study, the proportion of cutaneous adverse drug reactions was more common in housewives (32%). According to the study conducted by Swaroop et al., students (25%) were the most common group affected [5].

In our study, the majority of patients were from rural areas, 54% (n=108). This finding was similar to the study done by Swaroop et al. (56%) [5]. As there is a non-availability of specialist doctors in rural areas, the patient directly goes to the pharmacist and purchases medication for prompt relief. In contrast, a study conducted by Varshney et al. showed that the majority of patients were from urban areas (60.78%) [9].

In the current study, around half of the patients (49%; n=98) used creams for one to three months before developing cutaneous adverse drug reactions. This duration is due to acute effects of the triple combination, fixed drug combination, and other herbal irritant OTC agents used by patients in our study. In a study by Swaroop et al., most patients (29%) used creams for more than a year [5]. These studies mainly include cutaneous ADR of only topical corticosteroids, but we have included all types of topical medication in our study.

In our study, most patients (55.5%) had applied them twice daily. Varshney et al. detected similar results, showing that 84.05% of patients had used them twice daily [9].

Our study found the most common indication for use was fungal infection (76%; n=152). The reasons behind that are the high temperature and humidity in our region, the high prevalence of dermatophytic infections (20-25%) [11] in different parts of the country, and the high cost of antifungal therapy. Therefore, patients suffering from a chronic dermatophytic infection prefer to use OTC creams available from pharmacists instead of going to a dermatologist. This finding was similar to the study conducted by Varshney et al. [9], which showed fungal infection (59.5%) as the most common indication. The study by Birudala et al. showed that most patients used topical medications for acne (39.7%), which was different from our research [3].

**Figure 3:**
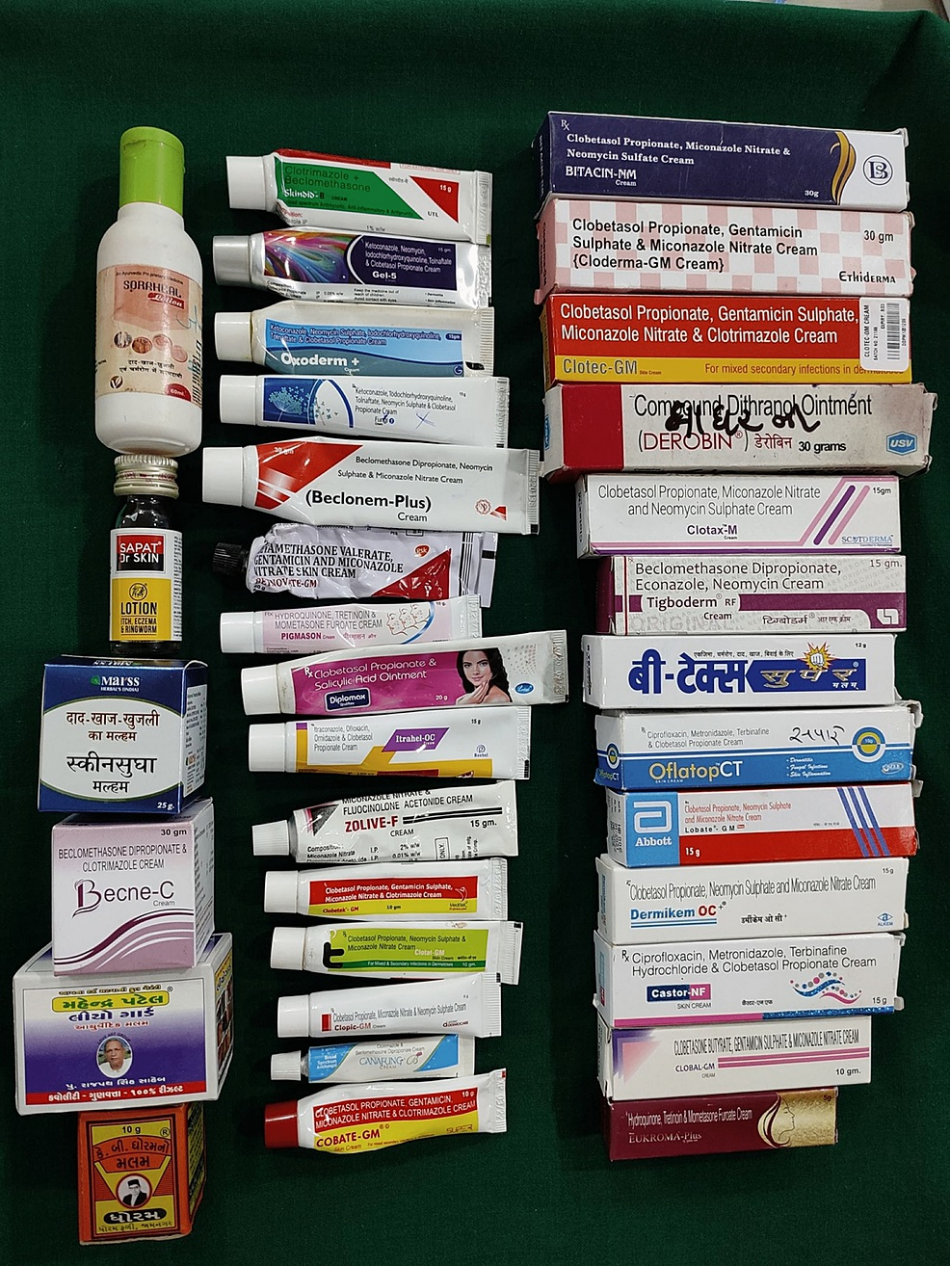
Various topical medicines misused by patients

In this study, the most commonly used creams were fixed-dose combinations containing antifungal, antibacterial, and steroid agents (40%; n=80). The field of dermatology is upgraded by the introduction of topical corticosteroids of various potencies due to their anti-inflammatory, anti-proliferative, immunosuppressive, anti-pruritic, melanopenic, anthropogenic, and vasoconstrictive properties on the skin. But flooding of these FDC products at the doorstep of patients, lack of knowledge of non-dermatologist doctors, Ayurvedic doctors, and Homeopathic doctors has resulted in the use of superpotent steroids for the wrong indication with the wrong method of application and for an extended period. Apart from these FDC creams, the triple combination containing mometasone furoate 0.1% + hydroquinone 2% + tretinoin 0.025% was used by 15% patients. Different types of OTC such as Derobin, salicylic acid, coal tar, Sapat lotion, Zalim, Skin Sudha, Ayushi, Parasmani, Soraheal, Dharam, KB Ghoram, and Mahendra Patel Malam were frequently used by 27.5% patients (Figure 3).

Unregulated marketing (even in the newspaper, hoardings, etc.) and selling different types of *malam* (ointment) has raised the number of cutaneous ADR. The results were consistent with a study conducted by Varshney et al., where 47.9% of patients misused fixed drug combinations containing steroid+ antibacterial+ antifungal creams [9].

**Figure 4:**
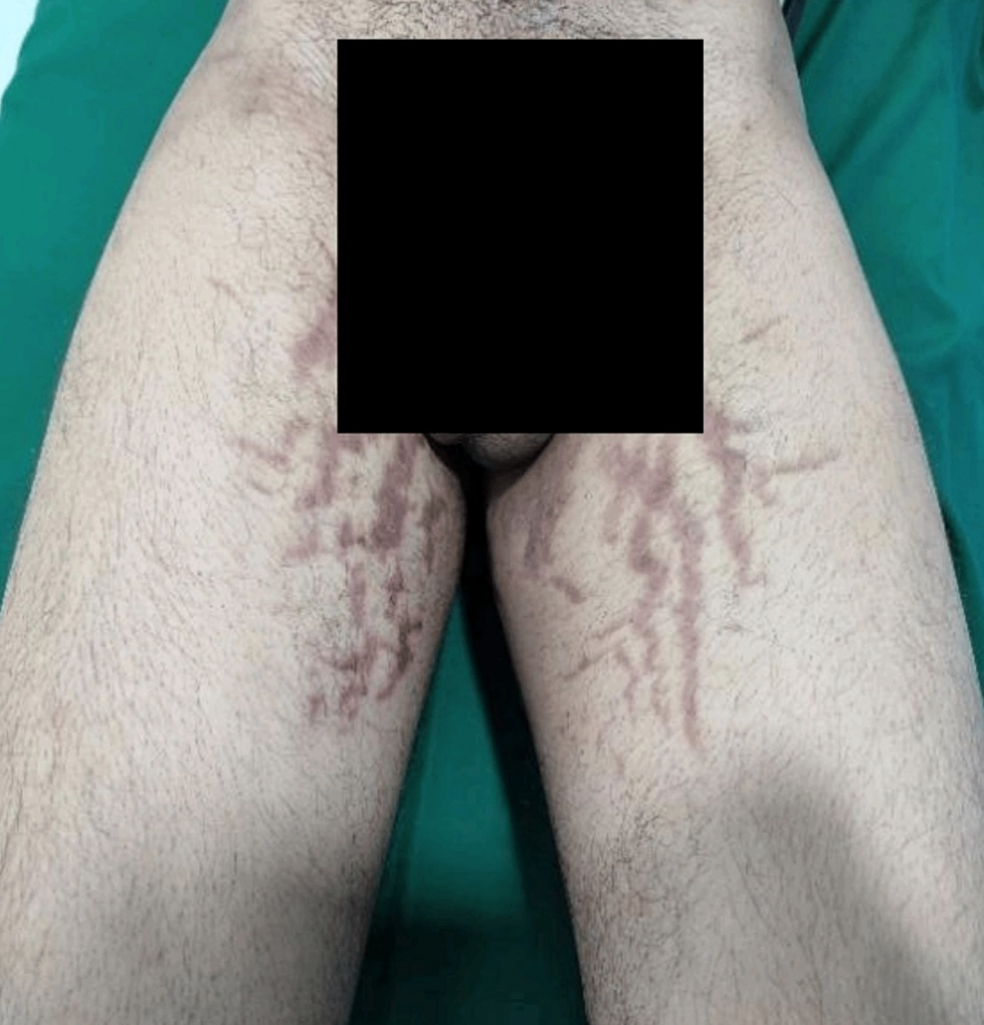
Striae rubra

The most common cutaneous adverse drug reaction detected in our study was tinea incognito 36% (n=72), followed by irritant contact dermatitis (12.5%; n=25). Figure 4 shows striae rubra developing in a patient after using FDC cream for Tinea infection.

**Figure 5:**
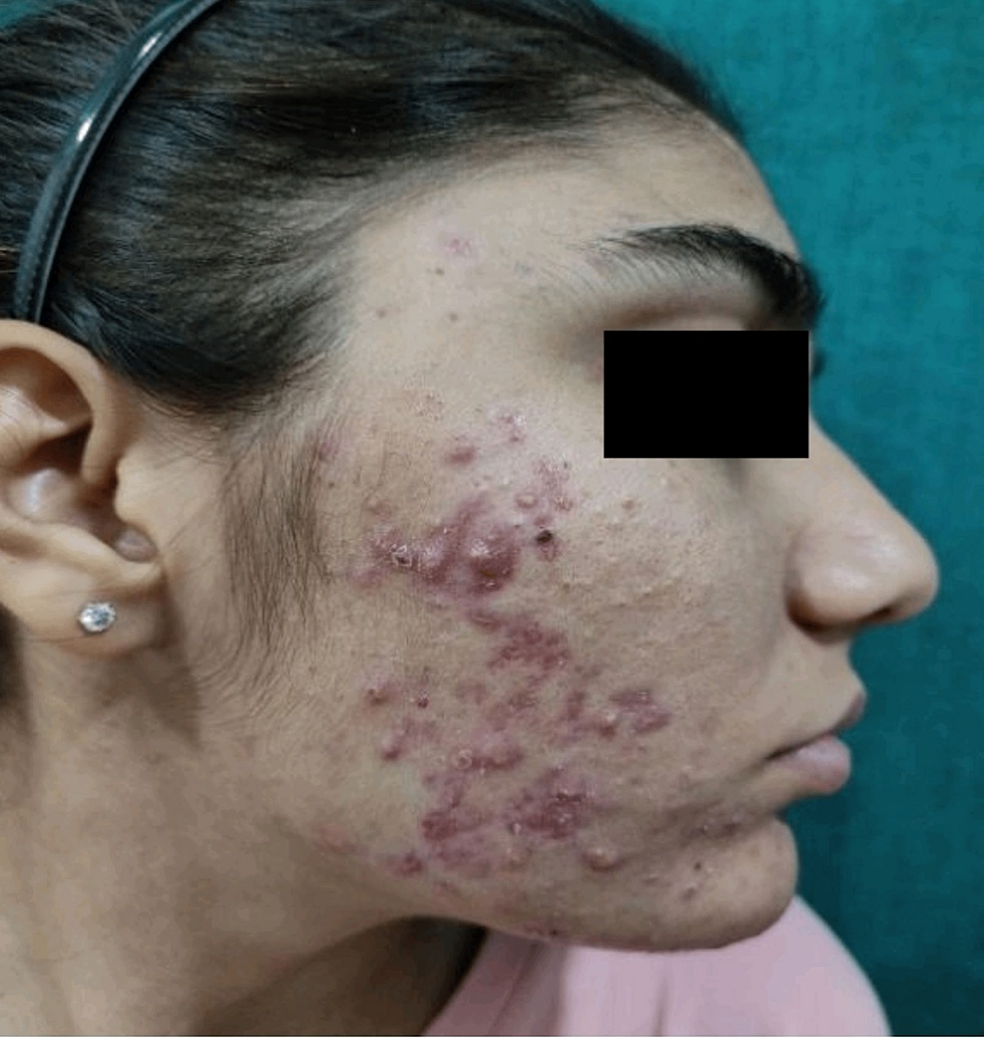
Acneiform eruption

Figure 5 shows a patient developing acneiform eruption after using a triple combination, which the patient applied for melasma.

**Figure 6:**
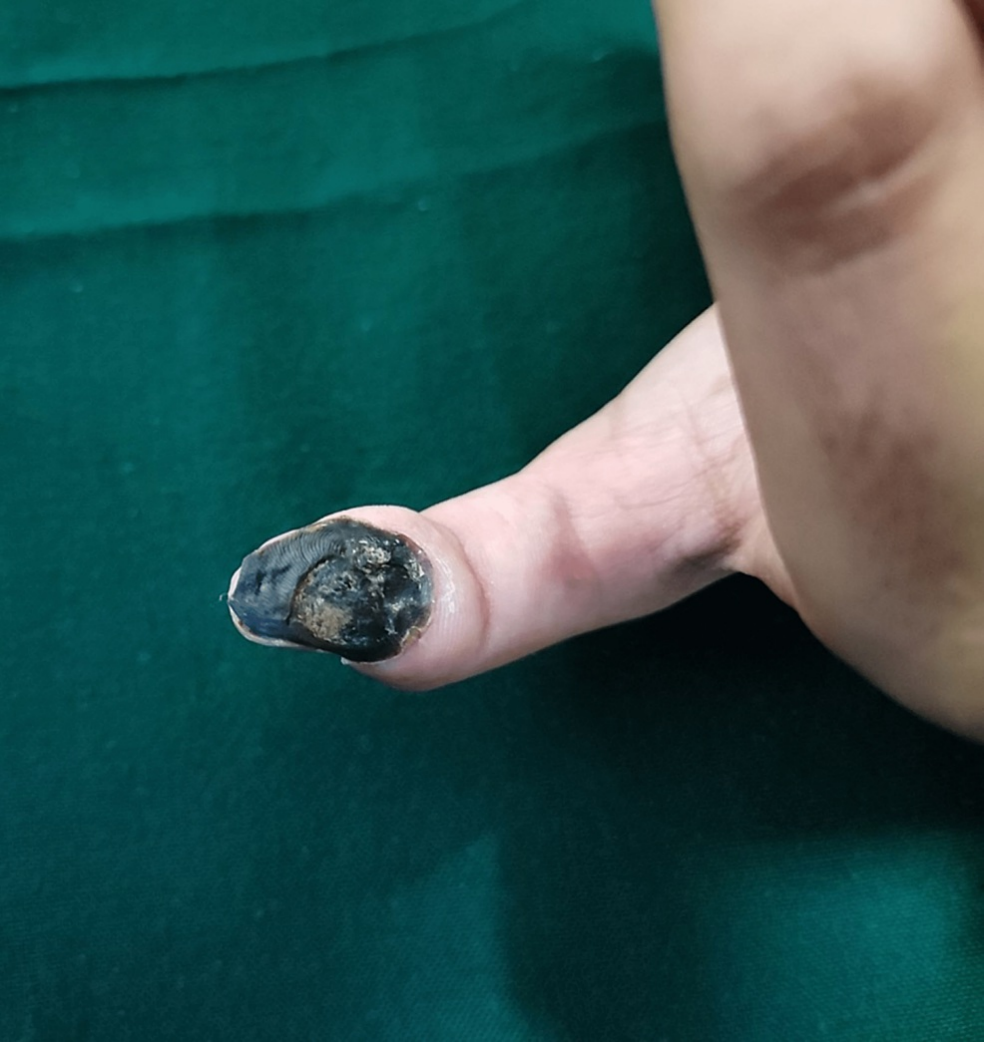
Necrotic change

Figure 6 shows necrotic changes in the right thumb after using Zalim Malam for fingertip eczema.

Meena et al. [6] and Varshney et al. [9] found similar results, in which tinea incognito was the most common cutaneous ADR, 49.46% and 41.1%, respectively. The emergence of resistance due to misuse of topical medication will ultimately lead to the requirement of a prolonged duration of treatment and an increase in the total cost of therapy.

In our study, half of the patients, 43% (n=86) received creams and ointments from pharmacists. This study observation was similar to the study conducted by Dabas et al. [7] in which 30.88% of patients had received creams or ointments from pharmacists. So awareness is required for pharmacists, the general population, and non-dermatologist doctors about the misuse and adverse effects of topical medication because they are unfamiliar with hazardous effects caused by the same.

The limitation of the study was the small sample size and short duration. Rechallenge with topical medicine was not done in our study. More numbers of studies involving pharmacists, general practitioners, and dermatologists from the private sector are required.

## Conclusions

This study shows that the misuse of topical medications is rampant in our community. Fixed drug combinations are most commonly misused topical medications. The most common indication of use was a fungal infection while tinea incognito and irritant contact dermatitis were the most commonly detected entities of cutaneous ADR. Topical medications marketed as herbal, safe, and claimed to be 100% effective are flooding the market, and they are the new emerging culprits for cutaneous ADR. There is an urgent requirement for strict regulations over the manufacturing, sales, and marketing of OTC topical medications to reduce the burden of cutaneous ADR in the community. Our data shows a need to create awareness among peer medical personnel, pharmacists, government authorities, and the general public regarding the rationale for using topical medicines.
